# Thermodynamic insights into histone transfer among chaperones

**DOI:** 10.1186/1756-8935-6-S1-P111

**Published:** 2013-04-08

**Authors:** Wallace H Liu, Sarah C Roemer, Alex M Port, Mair EA Churchill

**Affiliations:** 1 University of Colorado at Denver, Aurora, Colorado, USA

## Background

Together with non-histone proteins nucleosomes assemble the eukaryotic genome into higher order structures known as chromatin. Chromatin structure is dynamic, as it is continually assembled and disassembled for factors that carry out the processes of transcription, replication, DNA repair, and recombination to gain access to the DNA. The deposition of the histones H3/H4 onto DNA to give the tetrasome intermediate and the displacement of H3/H4 from DNA are thought to be first and last steps in nucleosome assembly and disassembly, respectively. Anti-silencing function 1 (Asf1) and Chromatin Assembly Factor (CAF-1) are chaperones of histones H3/ H4 that function together in the replication dependent chromatin assembly pathway.

## Materials and methods

In order to investigate the molecular basis of the activity of Asf1 and CAF-1 in chromatin assembly and disassembly, we employed a variety of methods, including X-ray crystallography, biophysical and biochemical methods using full-length proteins in the budding yeast system.

## Results

To understand the mechanism of histone H3/H4 transfer among Asf1, CAF-1, and DNA from a thermodynamic perspective, we measured the binding affinities for their various complexes. Asf1 has the ability to directly deposit H3/H4 dimers onto the DNA, but additional factors are required for their removal from DNA. The C-terminal tail of Asf1 greatly enhances the interaction of Asf1 with H3/H4 and with CAF-1. Surprisingly, although H3/H4 also enhances the interaction of Asf1 with the CAF-1 subunit Cac2, H3/H4 forms a tight complex with CAF-1 exclusive of Asf1, with an affinity weaker than Asf1-H3/H4 or H3/ H4-DNA interactions. Unlike Asf1, monomeric CAF-1 binds to multiple H3/H4 dimers, which ultimately promotes the formation of (H3/H4)_2_ tetramers on DNA.

## Conclusions

The H3/H4-Asf1 complex is a thermodynamic histone sink. The transition of H3/H4 from the Asf1-associated dimer to the DNA-associated tetramer is thermodynamically favored, but occurs through a less stable intermediate complex with CAF-1.

